# Biosafety of Non-Surface Modified Carbon Nanocapsules as a Potential Alternative to Carbon Nanotubes for Drug Delivery Purposes

**DOI:** 10.1371/journal.pone.0032893

**Published:** 2012-03-22

**Authors:** Alan C. L. Tang, Gan-Lin Hwang, Shih-Jung Tsai, Min-Yao Chang, Zack C. W. Tang, Meng-Da Tsai, Chwan-Yao Luo, Allan S. Hoffman, Patrick C. H. Hsieh

**Affiliations:** 1 Institute of Clinical Medicine, National Cheng Kung University & Hospital, Tainan, Taiwan; 2 Nano-Powder and Thin Film Technology Center, Industrial Technology Research Institute, Tainan, Taiwan; 3 Institute of Biomedical Engineering, National Cheng Kung University & Hospital, Tainan, Taiwan; 4 Center for Micro/Nano Science and Technology, National Cheng Kung University & Hospital, Tainan, Taiwan; 5 Department of Surgery, National Cheng Kung University & Hospital, Tainan, Taiwan; 6 Department of Bioengineering, University of Washington, Seattle, Washington, United States of America; 7 Institute of Biomedical Sciences, Academia Sinica, Taipei, Taiwan; University of California, Merced, United States of America

## Abstract

**Background:**

Carbon nanotubes (CNTs) have found wide success in circuitry, photovoltaics, and other applications. In contrast, several hurdles exist in using CNTs towards applications in drug delivery. Raw, non-modified CNTs are widely known for their toxicity. As such, many have attempted to reduce CNT toxicity for intravenous drug delivery purposes by post-process surface modification. Alternatively, a novel sphere-like carbon nanocapsule (CNC) developed by the arc-discharge method holds similar electric and thermal conductivities, as well as high strength. This study investigated the systemic toxicity and biocompatibility of different non-surface modified carbon nanomaterials in mice, including multi-walled carbon nanotubes (MWCNTs), single-walled carbon nanotubes (SWCNTs), carbon nanocapsules (CNCs), and C_60_ fullerene (C_60_). The retention of the nanomaterials and systemic effects after intravenous injections were studied.

**Methodology and Principal Findings:**

MWCNTs, SWCNTs, CNCs, and C_60_ were injected intravenously into FVB mice and then sacrificed for tissue section examination. Inflammatory cytokine levels were evaluated with ELISA. Mice receiving injection of MWCNTs or SWCNTs at 50 µg/g b.w. died while C_60_ injected group survived at a 50% rate. Surprisingly, mortality rate of mice injected with CNCs was only at 10%. Tissue sections revealed that most carbon nanomaterials retained in the lung. Furthermore, serum and lung-tissue cytokine levels did not reveal any inflammatory response compared to those in mice receiving normal saline injection.

**Conclusion:**

Carbon nanocapsules are more biocompatible than other carbon nanomaterials and are more suitable for intravenous drug delivery. These results indicate potential biomedical use of non-surface modified carbon allotrope. Additionally, functionalization of the carbon nanocapsules could further enhance dispersion and biocompatibility for intravenous injection.

## Introduction

The superior electrical and thermal conductivities, optical properties, and mechanical strength of carbon nanotubes (CNTs) and C_60_ fullerene (C_60_) make these nanomaterials ideal for use in structural supports, circuits, biosensors, batteries and solar cells [1], [2]. Different forms of fullerene have been envisioned as components of potential therapeutic devices in which they might act as tissue scaffolds 3, implants [4], biological microelectromechanical systems, biosensors, medical contrast agents, and drug delivery carriers [5]–[8]. Accordingly, the toxicology of CNTs has been widely investigated to understand the biological effects of these nanomaterials. Previous studies have demonstrated the *in vivo* toxicity and poor biocompatibility of multi-walled CNTs (MWCNTs), single-walled CNTs (SWCNTs) [9], [10] and C_60_ 11 following inhalation 12, intratracheal instillation 13 or intraperitoneal injection [15]–[18].

Nanomaterials have been investigated as a technology to deliver therapeutic agents within the body with the ability to bypass tough biological barriers 19. Like most nanomaterials, the dimensions of CNTs are on the nanoscale, providing a high surface-area-to-volume ratio for efficient drug conjugation or encapsulation. Because great interest in using fullerenes for drug delivery has been generated, different forms of these carbon nanomaterials have been developed. To effectively use these nanomaterials for drug delivery, the biocompatibility and toxicity of these nanomaterials within biological systems must be fully characterized and understood [20]. Several reviews and studies have reported the toxicity of unmodified MWCNTs, SWCNTs, and C_60_ [9]–. The van der Waals forces on the surfaces of pristine CNTs cause hydrophobic interactions between CNTs, resulting in unwanted aggregation, agglomeration and wiring[9], [18], [20]. To avoid excessive surface interactions and to decrease toxicity, studies have opted to cut and extensively surface-modify CNTs for enhanced biocompatibility [4]–[10], [15]–[18], [20]. Despite so, overwhelming toxicological reports of CNTs have given rise to the consensus that these long and rigid CNTs are not suitable for *in vivo* applications [10]. Though surface modifications do in fact reduce toxicity to certain degree, the extensive act of functionalization and related modifications is simply masking the root cause of toxicity of CNTs, derived from the material's surface.

Recently, carbon nanocapsules (CNCs) have emerged as a novel carbon-based nanomaterial synthesized in a manner similar to that used for CNTs and C_60_ [21], [22], providing comparable chemical composition, and electrical, thermal, and mechanical characteristics. Following the footsteps of CNTs, CNCs have also found success in different applications, including transceiver modules and photovoltaics [23], [24], [25]. In contrast, biomedical applications using CNCs have not yet been attempted. A major difference, namely the aspect ratio, exists in the spherical geometry of CNCs, compared to long, tangling characteristics of CNTs. Intuitively, the low aspect ratio structure of CNCs are more dynamically suitable for *in vivo* delivery. Herein, we investigate the *in vivo* biocompatibility of non-modified CNCs, MWCNTs, SWCNTs, and C_60_ in mice, providing insight into advantages of using carbon nanocapsules for systemic drug delivery.

## Materials and Methods

### Ethics Statement

All animal procedures were approved by the National Cheng Kung University Institutional Animal Care and Use Committee.

### Carbon nanomaterial preparation

The CNCs were prepared as described previously [21], [22]. Briefly, an inert gas (helium, argon, or nitrogen), was introduced into an arc chamber containing a graphitic cathode and anode. A current was then introduced to the chamber that had sufficient voltage (10–30 V) for a carbon arc reaction to take place. The rate of the inert gas was controlled to approximately 60 to 90 cm^3^/min, and the chamber pressure was maintained between 1 and 2 atm. A pulse current was used (50–70 Hz; 50–500 A), and the deposit on the cathode was collected and passed through a 0.22 μm filter for purification. The deposits contained roughly 70% CNCs before purification and at least 95% after purification. MWCNTs were produced in a similar manner using the arc-discharge method under an argon atmosphere, as previously described [23]. A direct current electric field was applied, and deposits were collected from the cathode and purified. The deposits were roughly 50% pure and became more than 95% pure after purification. SWCNTs were purchased from SES research (Texas, USA) and C_60_ from Sigma Aldrich (Missouri, USA). All carbon nanomaterials were dispersed in 1****wt % polyvinyl alcohol (PVA) at 5–10 mg/ml. Immediately before injection, the nanomaterial dispersions were sonicated for 1 h (E60H, Elma Ultrasonics, Germany).

### TEM analysis

Carbon nanoparticles were dispersed in 1% PVA onto Formvar/carbon-coated 200 mesh copper grids (Ted Pella Inc, CA, USA) for TEM analysis using an H-7500 TEM (Hitachi, Japan). The samples were lyophilized for 24 h and imaged by an experienced technician.

### Animal protocols and experiments

The investigation conformed to the *Guide for the Care and Use of Laboratory Animals* published by the US National Institutes of Health (NIH Publication No. 85–23, revised 1996). Adult FVB mice 8 to 12 weeks old and weighing 20 to 28****g were used in this study. The mice were anesthetized with sodium pentobarbital (50 mg/kg, i.p.). After the anesthetic had taken effect, the mice were injected with normal saline, 1% PVA, CNCs, C_60_, SWCNTs, or MWCNTs at a dose of 50, 25 or 12.5****μg/g bw via the tail vein, comparable to previous studies [26]–[28]. Normal saline and 1% PVA were injected at the same volume as the nanomaterials. Nanomaterials were injected at a diluted concentration so that each injection was approximately 200–250 μl (injection volume varied due to animal weight variations). A gauge was used to stop the bleeding of the tail, and the mice were allowed to recover from anesthesia in cages in a temperature- and climate-controlled environment with food and water. Mice were deeply anesthetized prior to sacrifice by cervical dislocation at different time points for organ harvesting and urine and blood collection. Blood collection was performed by cardiac puncture prior to cervical dislocation.

### Mouse survival study

Mice were separated into three dosage groups with 11 or 12 mice in each group. The carbon nanomaterials were sonicated for 1 h prior to injection, and a total volume of 200–250 μl was injected into each mouse (injection volume varied due to animal weight variations). Nanomaterials were diluted with 1% PVA to maintain similar injection volumes for all three doses. After injection, mice were returned to their cages to recover from the anesthesia in a temperature- and climate-controlled environment. The mice were monitored closely for the first 6 hours, at 12 hours, and then every day following the first day. The time of death of each mouse was recorded, and at the end of 7 d, the mice were sacrificed, and their organs were harvested for tissue sectioning.

**Figure 1:**
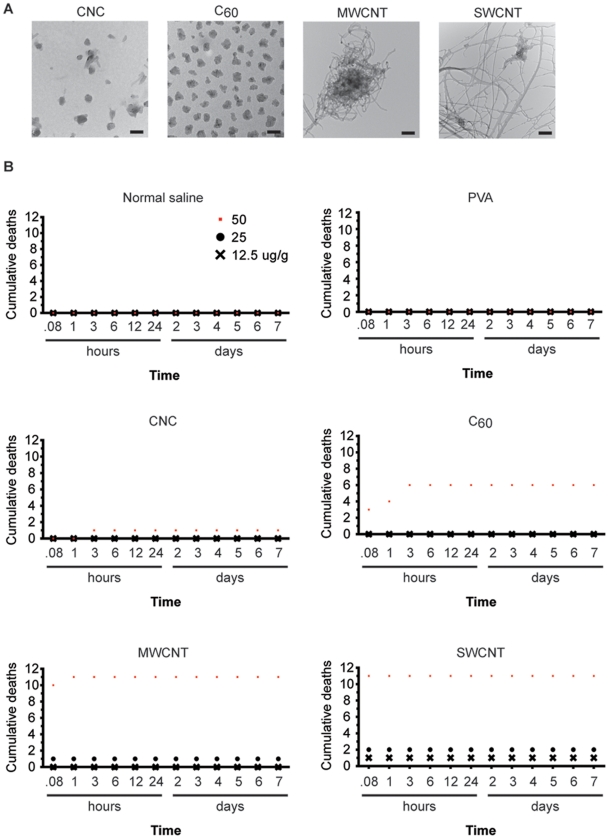
Mouse survival curves after carbon nanomaterial injection. (**A**) TEM analysis of carbon nanocapsules (CNCs), C_60_ fullerene (C_60_), multi-walled carbon nanotubes (MWCNTs), and single-walled carbon nanotubes (SWCNTs) dispersed in 1% polyvinyl alcohol (PVA). SWCNTs formed large networks, and MWCNTs aggregated compactly. CNCs were well dispersed in PVA, while C_60_ aggregated to size as large as CNCs. The scale bar is 100****nm. (**B**) Cumulative deaths of mice intravenously injected with different doses of carbon nanomaterials. SWCNTs and MWCNTs had the highest toxicity, which was dose dependent, decreasing as the dose of the carbon nanomaterials decreased. No mortality was observed among the CNC-treated mice at 25 μg/g b.w. n = 12 for CNC, and C_60_ injected mice. n = 11 for NS, PVA, MWCNT, and SWCNT injected mice. Red square, 50 μg/g; black dot, 25 μg/g; black cross, 12.5 μg/g.

### Tissue sections and nanomaterial retention quantification

Organs were harvested 6 h or 7 d after injection with the nanomaterials, washed in PBS and fixed in 4% paraformaldehyde at 4°C overnight. The organs were then stored in 70% ethanol prior to paraffin embedding. Sections were stained with hematoxylin and imaged using an Axio Scope A1 imaging system (Carl Zeiss). For the nanomaterial retention study, at least 2 tissue sections from each lung were used and were left unstained to reduce background and false positive signals. Whole tissue sections were imaged by HistoFAXS (TissueGnostics, Austria) at a 200x final magnification and were analyzed with HistoQuest (TissueGnostics, Austria) for automated structure detection, automatic color separation, and quantification.

### Inflammatory cytokine study

Blood was collected from mice 6 hours post-injection and was allowed to sit at room temperature for at least 1 h. Lipopolysaccharide (LPS) injected intravenously served as positive control (5 mg/kg, sigma). Samples were centrifuged at 1500 g for 10 minutes to obtain serum. Serum samples were then analyzed using ELISA kits for mouse IL-1β and mouse IL-6 for the detection of cytokines. These assays were performed according to the manufacturer's instructions (AssayPro, USA). Normal saline was used as a negative control, and lipopolysaccharide was used as a positive control. Lung tissues were also collected 6 hours post-injection and homogenized in 500****μl of lysis buffer containing protease inhibitors. Homogenized samples were centrifuged at 14,000****RPM for 20 minutes to remove debris. The supernatants were analyzed using ELISA kits for mouse IL-1β and mouse IL-6 for the detection of cytokines. Cytokine levels were normalized to the total protein level determined by the BCA assay (Pierce, USA).

### Statistical Analysis

All data are presented as the mean±sem. Data were analyzed by one-way ANOVA followed by Tukey's post hoc test using Prism 5 (GraphPad, USA). A value of *P*<0.05 was considered statistically significant.

## Results

### Physical characteristics of carbon nanomaterials

Non-modified CNTs, whether single-walled (SWCNT) or multi-walled (MWCNT), form networks and aggregates even when dispersed in a surfactant such as PVA (Fig. 1a). The tube diameter of MWCNT were approximately 25 nm measured from TEM images. SWCNT diameters ranged from 2 nm to 25 nm (Table S1). C_60_, though much more uniform and dispersed than CNTs, still aggregated, forming 100 nm in diameter clusters. C_60_ molecules have a very low solubility and an extremely high density. Therefore, these nanoparticles settle within minutes even when dispersed in PVA after sonication and mixing (Figure S1). CNCs were much more uniformly dispersed and each particle was approximately 50 nm in diameter (Fig. 1a).

**Figure 2:**
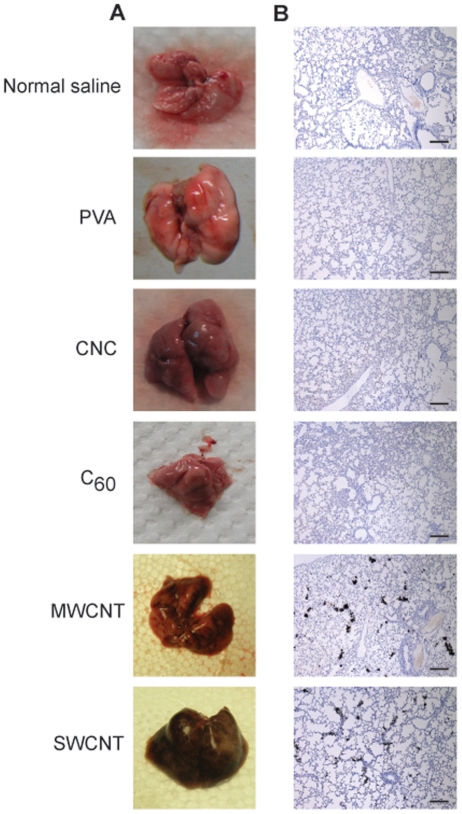
Lung tissues and lung tissue sections after carbon nanomaterial injection. (**A**) Excised lungs 10 min after mice were injected with 50 μg/g b.w. of different carbon nanomaterials. (**B**) Lung tissue sections 10 min after mice were injected with 50 μg/g b.w. of different carbon nanomaterials. Mice receiving C_60_ fullerene (C_60_), multi-walled carbon nanotubes (MWCNTs), and single-walled carbon nanotubes (SWCNTs) died within 10 minutes, and only mice in the carbon nanocapsule (CNC), normal saline, and polyvinyl alcohol (PVA) groups had to be sacrificed. Tissue sections were stained with hematoxylin. The scale bar is 400 μm.

### In vivo toxicity of carbon nanomaterials and cause of death

To study the *in vivo* toxicity of carbon nanomaterials, different carbon nanomaterials were intravenously injected into mice at three different doses. Strikingly, none of the mice receiving 50 µg/g b.w. of either MWCNTs or SWCNTs survived (n = 11 for each). By contrast, mice injected with CNCs had a 91.7% survival rate (n = 12), while half of the mice injected with C_60_ died (6 out of n = 12; Fig. 1b). The toxicities of the nanomaterials were dose dependent, as shown by the survival curves for the three different doses (Fig. 1b). Interestingly, all of the mice injected with 25 and 12.5 µg/g b.w. of CNCs survived, while some of the mice injected with these doses of MWCNTs or SWCNTs died (Fig. 1b).

**Figure 3:**
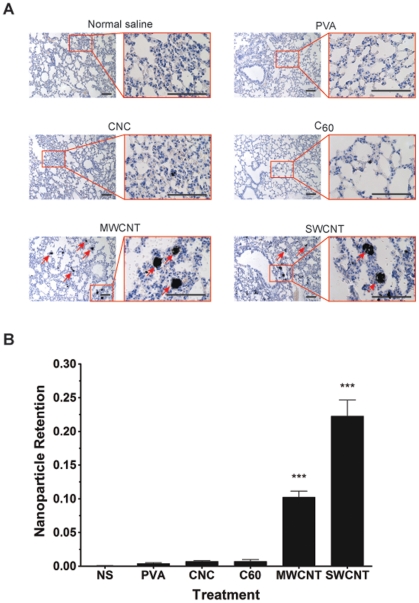
Carbon nanomaterial retention in the lungs. (**A**) Lung tissue sections of mice 7 days after intravenous injection with carbon nanomaterials at 25 μg/g b.w. (**B**) Automatic carbon nanomaterial retention quantification in the lungs. High-magnification images (red-bordered images) show large carbon nanotube aggregates blocking the blood vessels of the lungs (arrows). SWCNTs and MWCNTs were retained in the lungs at much higher rates compared to CNCs or C_60_. Tissue sections were stained with hematoxylin. Scale bar = 100 μm. ***P<0.0001 compared to CNCs and C_60_, n = 4 in all groups. NS, normal saline; PVA, polyvinyl alcohol; CNCs, carbon nanocapsules; C_60_, C_60_ fullerene; MWCNTs, multi-walled carbon nanotubes; SWCNTs, single-walled carbon nanotubes.

Postmortem inspections of the mice receiving 50 µg/g doses revealed that CNTs were clearly visible in the lungs (Fig. 2a). As expected, the MWCNT- and SWCNT-injected groups had the darkest lungs, which were fully covered with black spots. Lungs from CNC- and C_60_-injected mice generally exhibited a pink hue similar to that of the normal saline and 1% PVA in deionized H_2_O (PVA) groups, both of which served as controls (Fig. 2a). Tissue sections of these lungs further revealed that a large surface area of MWCNT and SWCNT lungs was occupied by CNTs (Fig. 2b).

**Figure 4:**
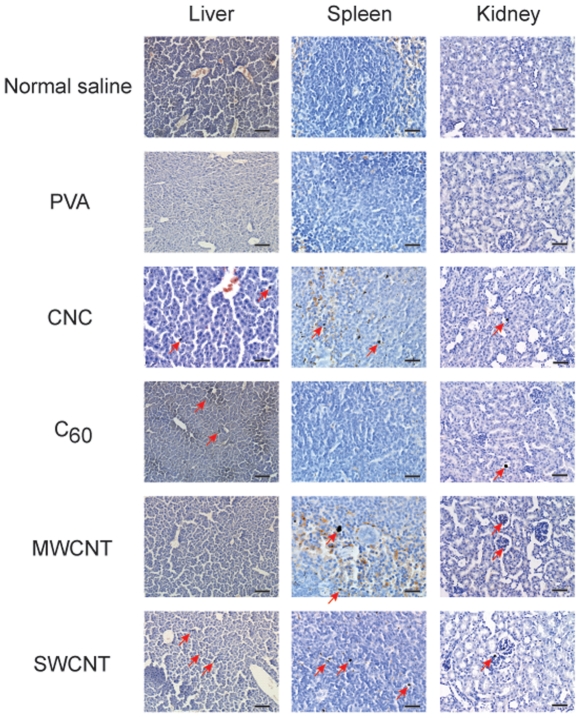
Carbon nanomaterial retention in vital organs. Liver, spleen, and kidney tissue sections of mice 7 days after intravenous injection with carbon nanomaterials at 25 μg/g b.w. Carbon nanomaterials are indicated by arrows. Tissue sections were stained with hematoxylin. The scale bar is 50 μm. PVA, polyvinyl alcohol; CNCs, carbon nanocapsules; C_60_, C_60_ fullerene; MWCNTs, multi-walled carbon nanotubes; SWCNTs, single-walled carbon nanotubes.

**Figure 5:**
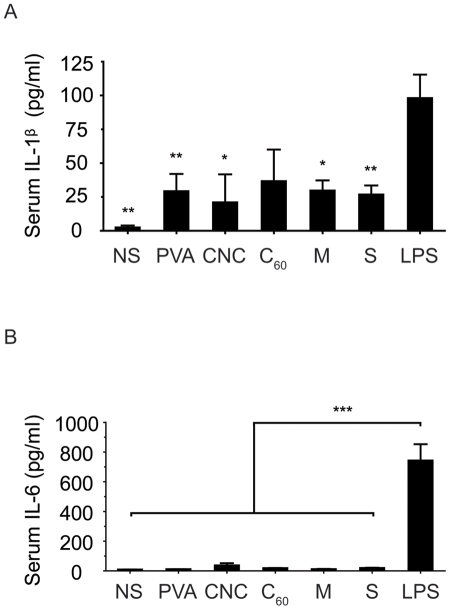
Systemic inflammatory cytokine level in mice. Serum and lung tissue IL-1β and IL-6 levels 6 hours post-injection with carbon nanomaterials. There is no significant difference between all groups (except LPS). n = 5, *P* = 0.0029 (serum, IL-1β); n = 5, *P* = 0.0001 (serum, IL-6); n = 4. *P<0.05, **P<0.01, ***P<0.001 significantly different compared with the LPS group. NS, normal saline; PVA, polyvinyl alcohol; CNCs, carbon nanocapsules; C_60_, C_60_ fullerene; M, multi-walled carbon nanotubes; S, single-walled carbon nanotubes.

### Biodistribution and retention of carbon nanomaterials

To quantify and compare the retention of the carbon nanomaterials in the lungs, surviving mice from the 25 µg/g dose injections were sacrificed on day 7, and the lungs were collected for tissue section analysis. Similar to injections of 50 µg/g, lung tissue sections from mice injected with 25 µg/g showed a similar trend in the lung retention of the nanomaterials. MWCNTs and SWCNTs were widely distributed and accumulated in the lungs, while CNCs and C_60_ were scarce (Fig. 3a). Automated microscopic whole tissue section analysis revealed that MWCNTs retained in the lungs by more than a factor of 14 compared to CNCs or C_60_, while SWCNTs retained by a factor of more than 30 (Fig. 3b). Because CNTs formed larger aggregates in the range of 200–1000 nm (Fig. 1), more blood vessels were observed to be have been clogged by MWCNTs and SWCNTs (Fig. 3a). By contrast, CNCs and C_60_ did not form aggregates larger than 200 nm; therefore, they passed through to other organs including the liver, spleen, and kidney, or cleared through the renal system (Fig. 4, Figure S2). SWCNTs were lethal even at the dose of 12.5 µg/g following systemic injection, and retention was found to be twice that of MWCNTs in the lungs.

**Figure 6:**
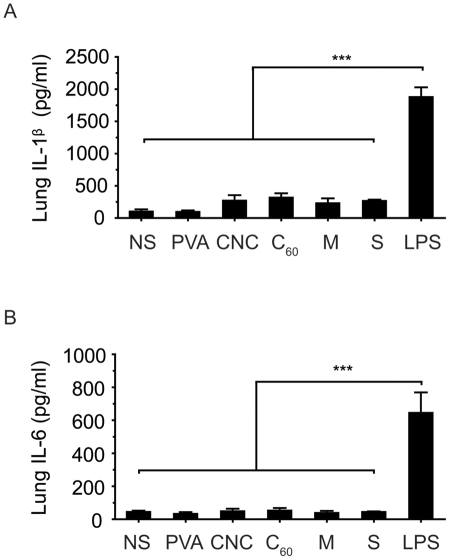
Lung tissue inflammatory cytokine level in mice. Lung tissue IL-1β and IL-6 levels 6 hours post-injection with carbon nanomaterials. There is no significant difference between all groups (except LPS). Lung-tissue cytokine levels were normalized to the total protein level determined using a BCA kit (Pierce, USA). n = 5, ***P = 0.0001 (lung tissue, IL-1β); n = 4, *P* = 0.0001 (lung tissue, IL-6), compared to the LPS group. NS, normal saline; PVA, polyvinyl alcohol; CNCs, carbon nanocapsules; C_60_, C_60_ fullerene; M, multi-walled carbon nanotubes; S, single-walled carbon nanotubes.

### Systemic inflammatory response after IV injection of carbon nanomaterials

Although CNTs and nanoparticles have the potential to be used for drug delivery applications, the foreign body reaction of nanomaterials is a concern. Nanotoxicity arises from the inflammatory responses to foreign bodies, cellular uptake and inflammatory cytokine production, among many other acute responses [29], [30]. Interleukin–1 beta (IL-1β) and interleukin–6 (IL-6) are two important inflammatory cytokines induced during inflammation that mediate the inflammatory response. To examine the acute systemic response after injecting the nanomaterials, serum was collected from the surviving mice for an ELISA analysis. Lipopolysaccharide (LPS) served as a positive control for inducing systemic inflammatory cytokines. Serum from all groups of injections showed no significant difference with each other in IL-1β and IL-6 expression levels (Fig. 5). Furthermore, the cytokine levels in the lung tissue samples also showed the same result (Fig. 6). LPS injected samples, both serum and lung tissue, were significantly were significantly higher in both IL-1β and IL-6. Pellets from lung-tissue preparation for cytokine detection revealed consistent results from tissue sections (Figure S3). MWCNT and SWCNT lung homogenates were extremely dark while CNC and C_60_ groups were much lighter in color.

## Discussion

Although nanoscopic in feature size, pristine CNT surfaces strongly attract each other through van der Waals forces, causing aggregation and network formation [5], [9], [18], [20]. This non-dispersing interaction prevents CNT from being an ideal tool for drug delivery through intravenous injection [18], [20]. Consistent with previous studies, we found that CNTs were prone to aggregate and easily bundled on itself [10]. To overcome the challenging aggregate-forming surface properties of CNTs, previous studies have put effort in cutting, functionalizing, and surface modifying CNTs [4]–[10], [15]–[18], [20]. However, surface modification is rather masking an existing flaw of CNT toxicity instead of actually eliminating this shortcoming. Most studies using CNTs for drug delivery purposes require cutting to reduce the overall length. However, in this form, CNTs are still too long and rigid drug delivery purposes [10]. Furthermore, different fabrication protocols introduce defects as part of the process, whether intentionally or as a side effect [31], [32]. These structural defects have been linked to causing acute lung toxicity, genotoxicity, and inflammatory responses [33]–[36]. Recently, CNCs have been produced using a method similar to that used for preparing CNTs using a pulsed plasma arc discharge method [21], [22]. Much like CNTs, CNCs have high electrical conductivity, thermal conductivity, strength, and surface-area-to-volume ratio [37]. However, CNCs differ from CNTs in that CNCs inherently have a much lower aspect ratio at around 1.5. Furthermore, CNCs are uniformly synthesized nanoparticles ranging from 40–60 nm while highly dense C_60_ form clusters up to 100 nm. Due to the previously mentioned properties, CNCs, unlike CNTs and C_60_, lack aggregating properties, which are much more favorably biocompatible for drug delivery purposes.

In our study, CNTs in the lungs formed aggregates of approximately the same diameter as the blood vessels and were trapped in a manner similar to a pulmonary embolism, clogging the blood vessels. Thus, the main cause of death of the mice injected with high dose CNTs is attributed to the mechanical obstruction of the blood vessels in the lungs, possibly leading to acute heart failure. By contrast, very few carbon nanomaterials were found in the CNC or C_60_ lung tissue sections. The CNC and C_60_ nanoparticles that remained in the lungs were extremely small, and most of these small nanoparticles may have escaped the highly vascularized lungs and traveled to other organs, while the aggregated MWCNTs and SWCNTs were easily trapped in the lungs. Consistent with previous reports, large aggregates of CNTs are the main concern for the biosafety of this material [20]. What remains unclear, however, is why there was high toxicity after the C_60_ injections despite the high clearance rate and the lack of an immune response. Lung and other vital organ tissue sections of the C_60_ group did not show any retention at any dose. Although C_60_ was absent from our tissue sections, we cannot rule out C_60_ retention due to the extreme small dimensions of this nanoparticle. Indeed, a recent study quantified C_60_ retention following intravenous injection in rats using liquid chromatography [38]. Their results show high acute retention of the particles in the vital organs, particularly the lung one day following injection, which decreased over the course of 4 weeks. Together with other studies that have reported C_60_ toxicity, it is reasonable that C_60_ fuller is lethal following intravenous injection.

The inflammatory response was consistent with other studies that CNTs or other forms of fullerene did not elicit an inflammatory response in any tissue, although the nanomaterials were distributed throughout the animal [20]. Both systemic and local tissue studies showed that cytokine levels were at the same level as normal saline treated groups at the acute phase. LPS groups were significantly higher in both IL-1β and IL-6 levels. Long-term studies are required to further understand whether these nanomaterials trigger chronic inflammation. However, similar to our findings, a recent study by Burke *et al* provided similar insight into the toxicity profile of CNTs [39]. MWCNTs injected at similar dosage were lethal in the acute stage, mainly by obstructing blood vessels in the lung. Furthermore, non-functionalized MWCNTs increased vWF and D-dimer levels following systemic injection, and reduced platelet count. Additionally, functionalization is effective in attenuating coagulation effects of MWCNTs.

To our knowledge, there has not been a single study that had comprehensively investigated the *in vivo* effect of different carbon nanomaterials injected intravenously. Most past studies aimed to characterize production plant safety by studying the pulmonary toxicity of carbon nanomaterials encountered through intratracheal instillation [9]. In addition, we included a novel carbon allotrope, CNC in this study. Here, we investigated the *in vivo* toxicity of raw, non-modified carbon nanomaterials delivered intravenously, hoping to gain an understanding of the dynamics of these nanomaterials *in vivo*. Nanotechnology has offered a wealth of possibilities for enhanced drug therapy to deliver therapeutic treatment. However, nanotoxicity must be well characterized before this technology can be used safely and effectively. Our results show that although certain properties of CNTs have allowed propelled this material to succeed in applications in sensors, circuitry, and structural components, the aggregating property of CNTs inhibit safe usage in drug delivery.

In this preliminary study, CNCs have been shown to be more biocompatible following intravenous injection and may emerge in the future for drug delivery purposes. These CNCs have already been functionalized to further enhance dispersion rates. In the future, these functionalized CNCs represent a novel carbon allotrope as a solution to the aggregating issue of CNTs, providing an alternate research opportunity towards drug delivery. Using current established methods, crosslinkers can be employed to conjugate antibodies, proteins, peptides, or small molecules onto functionalized CNCs for drug delivery purposes. We envision CNCs as a potential alternative to CNTs in the application of intravenous drug delivery.

## Supporting Information

Figure S1 **Carbon nanomaterial dispersions before and after sonication and shaking.** SWCNTs, MWCNTs, and CNCs better dispersed after sonication. C60 was too dense for sonication to have an effect. All nanomaterial dispersions were both sonicated and hand-shaken prior to injections. CNCs, carbon nanocapsules; C60, C60 fullerene; MWCNTs, multi-walled carbon nanotubes; SWCNTs, single-walled carbon nanotubes.
(TIF)

Figure S2 **Urine collected from mice 6 hours post-injection with carbon nanomaterials at 25 μg/g.** Urine samples revealed that some CNCs, C60, and MWCNTs can be cleared from the body as soon as 6 hours post-injection. No evidence of clearance of SWCNTs was observed throughout the study. PVA, polyvinyl alcohol; CNCs, carbon nanocapsules; C60, C60 fullerene; MWCNTs, multi-walled carbon nanotubes; SWCNTs, single-walled carbon nanotubes.
(TIF)

Figure S3 **Lung homogenate lysate after centrifugation at 14,000 rpm for 20 minutes. SWCNT and MWCNT lysates were much darker than those of all other groups.** PVA, polyvinyl alcohol; CNCs, carbon nanocapsules; C60, C60 fullerene; MWCNTs, multi-walled carbon nanotubes; SWCNTs, single-walled carbon nanotubes.
(TIF)

Table S1 **Table comparing physical properties of the different nanomaterials used in the study.**
(DOC)
