# Supplementary figures and images for: Biosafety of Non-Surface Modified Carbon Nanocapsules as a Potential Alternative to Carbon Nanotubes for Drug Delivery Purposes
Source: PLoS One. 2012 Mar 22;7(3):e32893. doi: 10.1371/journal.pone.0032893 (PMC3310837; doi:10.1371/journal.pone.0032893)

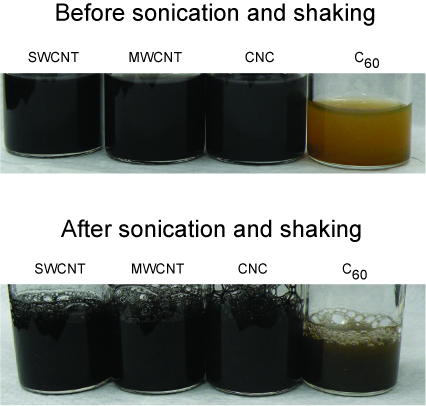

Supplement: Figure S1 — Carbon nanomaterial dispersions before and after sonication and shaking. SWCNTs, MWCNTs, and CNCs better dispersed after sonication. C60 was too dense for sonication to have an effect. All nanomaterial dispersions were both sonicated and hand-shaken prior to injections. CNCs, carbon nanocapsules; C60, C60 fullerene; MWCNTs, multi-walled carbon nanotubes; SWCNTs, single-walled carbon nanotubes. (TIF) [file pone.0032893.s001.tif]

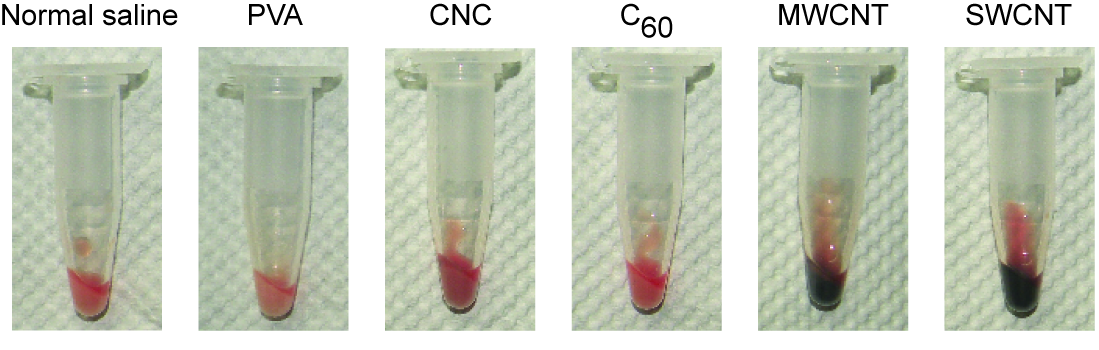

Supplement: Figure S2 — Urine collected from mice 6 hours post-injection with carbon nanomaterials at 25 μg/g. Urine samples revealed that some CNCs, C60, and MWCNTs can be cleared from the body as soon as 6 hours post-injection. No evidence of clearance of SWCNTs was observed throughout the study. PVA, polyvinyl alcohol; CNCs, carbon nanocapsules; C60, C60 fullerene; MWCNTs, multi-walled carbon nanotubes; SWCNTs, single-walled carbon nanotubes. (TIF) [file pone.0032893.s002.tif]

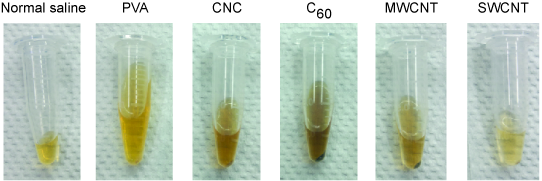

Supplement: Figure S3 — Lung homogenate lysate after centrifugation at 14,000 rpm for 20 minutes. SWCNT and MWCNT lysates were much darker than those of all other groups. PVA, polyvinyl alcohol; CNCs, carbon nanocapsules; C60, C60 fullerene; MWCNTs, multi-walled carbon nanotubes; SWCNTs, single-walled carbon nanotubes. (TIF) [file pone.0032893.s003.tif]
