# Supplementary material for: Biosafety of Non-Surface Modified Carbon Nanocapsules as a Potential Alternative to Carbon Nanotubes for Drug Delivery Purposes
Source: PLoS One. 2012 Mar 22;7(3):e32893. doi: 10.1371/journal.pone.0032893 (PMC3310837; doi:10.1371/journal.pone.0032893)
Supplement: Table S1 — Table comparing physical properties of the different nanomaterials used in the study. (DOC) [file pone.0032893.s004.doc]

**Supplementary Table 1**

**Supplementary Table 1**

**Comparison of the physical properties of the different carbon nanomaterials**

|  | CNCs^a^ | C60^b^ | MWCNTs^c^ | SWCNTs^d^ |
| --- | --- | --- | --- | --- |
| Diameter* (nm) | 52.4 ± 10.0 | 1 | ~25 | <2 |
| Aspect ratio | 1.5 ± 0.5 | 1 | >>2 | >>2 |
| Length (μm) | --- | --- | >>0.5 | 1–5 |

(a) Values calculated by TEM using three separate views and represented as mean

± standard deviation. (b) Theoretical values given. (c) Measured under TEM. (d)

Values obtained from the manufacturer * Outer diameter measurement.

CNCs, carbon nanocapsules; C60, C60 fullerene; MWCNTs, multi-walled carbon

nanotubes; SWCNTs, single-walled carbon nanotubes.
